# Effectiveness of Focal Extracorporeal Shockwave Therapy for Idiopathic Frozen Shoulder at 12‐Month Follow‐Up

**DOI:** 10.1155/aort/9822934

**Published:** 2026-11-01

**Authors:** Alfonso Di Giorno, Giovanni Carlisi, Valentina Sanzo, Giorgio Gasparini, Fabio Catani, Giuseppe Porcellini, Salvatore Maria Tecce, Stefano Gumina, Michele Mercurio

**Affiliations:** ^1^ CKF Di Giorno Excellence Medical Centre 40121 Bologna Italy; ^2^ Department of Orthopaedic and Trauma Surgery “Mater Domini” University Hospital “Magna Græcia” University V.le Europa 88100 Catanzaro Italy unicz.it; ^3^ Department of Orthopaedic and Trauma Surgery “Giovanni Paolo II” Hospital V.le Arturo Perugini 88046 Lamezia Terme Italy; ^4^ Research Center on Musculoskeletal Health, MusculoSkeletal Health@UMG Magna Graecia University 88100 Catanzaro Italy unicz.it; ^5^ Department of Orthopaedics and Traumatology Policlinico di Modena University of Modena and Reggio Emilia 41124 Modena Italy unimore.it; ^6^ Department of Orthopaedics and Traumatology “Policlinico Tor Vergata” Foundation 00133 Rome Italy; ^7^ Department of Anatomical, Histological, Forensic Medicine and Orthopaedics Sciences Sapienza University of Rome 00185 Rome Italy uniroma1.it

**Keywords:** Constant–Murley score, disability of the arm, focal extracorporeal shockwave therapy, idiopathic frozen shoulder, shoulder and hand (DASH), Visual Analog Scale (VAS)

## Abstract

The optimal treatment for frozen shoulder remains a subject of debate, within the orthopedic and rehabilitative communities, primarily due to the complexity of the pathology and the lack of consensus on therapeutic protocols, which range from conservative physical therapy to invasive surgical intervention. In this clinical landscape, noninvasive modalities have gained considerable traction for their potential to accelerate functional recovery and alleviate debilitating pain. The aim of this study was to evaluate the 12‐month long‐term results of focal extracorporeal shockwave therapy (f‐ESWT) in the treatment of idiopathic frozen shoulder. We prospectively collected data from 77 patients (42 females, 35 males; mean age: 57 years) suffering from idiopathic frozen shoulder between January 2021 and September 2022. The median number of f‐ESWT sessions performed was 6 (range: 2–10). Forty‐three patients received f‐ESWT treatment within 3 months of symptom onset. Patients were assessed at baseline, at the end of treatment, and at 2‐, 6‐, and 12‐month follow‐up intervals using the Constant–Murley Score (CMS), the Disabilities of the Arm, Shoulder, and Hand (DASH) questionnaire, and the Visual Analog Scale (VAS). A statistically significant improvement of CMS and reduction of DASH and VAS were observed from the baseline values to posttreatment evaluations (*p* < 0.001). Serial measurements revealed a bimodal trend of clinical improvements: a significant statistical difference from baseline values to both the end of the treatment and at the 2‐month follow‐up, followed by a clinical plateau up to a final, statistically significant improvement at the 12‐month follow‐up. Patients who initiated f‐ESWT within 3 months of symptom onset, as well as those who underwent fewer than seven sessions, exhibited significantly lower DASH values at the 2‐month follow‐up (*p* = 0.013). In conclusion, satisfactory clinical outcomes of f‐ESWT for idiopathic frozen shoulder can be expected at 12 months, particularly for patients starting treatment early (within 3 months of onset) and receiving fewer than seven sessions.

## 1. Introduction

From the earliest definition of periarthritis in the 19th century, the term “frozen shoulder” was later introduced by Codman in 1934 to describe a clinical condition characterized by shoulder pain, limited passive and active range of motion (ROM) of the glenohumeral joint [1–3], and functional disability [4–6].

The prevalence of frozen shoulder ranges between 2% and 5% in the general population, with symptoms typically appearing between 40 and 60 years of age [7, 8]. A higher likelihood has been reported for female patients with diabetes mellitus, thyroid disorders, stroke, myocardial infarction, inflammatory diseases, or history of prolonged immobilization [9–11]. The etiology remains poorly understood; it is often idiopathic, involving an inflammatory pattern that leads to contracture of joint capsule and the rotator interval. Frozen shoulder typically progresses through three phases [12, 13]: (i) the freezing phase, characterized by pain (often nocturnal) without initial loss of ROM, lasting 10–36 weeks [13, 14]; (ii) the frozen phase, lasting 4–12 months, featuring persistent pain and a significant loss of ROM [13, 14]; and (iii) the thawing (regression) phase where ROM progressively improves [13–15].

In most cases, the disease is self‐limited, though persistent symptoms remain in some patients [8, 16]. Generally, the goal is nonoperative therapy, reserving surgery for refractory cases. First‐line treatment for all stages of the frozen shoulder is the physical therapy, alone or in combination [17, 18]. Common protocols include mobilization, stretching, proprioceptive neuromuscular facilitation (PNF) supervised by physiotherapist, and home exercises [19, 20]. Nonsteroidal anti‐inflammatory drugs (NSAIDs) and corticosteroid injections are frequently used to mitigate pain and inflammation, improving patient tolerance for physical therapy [15, 21]. In this context, various conservative and minimally invasive approaches have been proposed, including thermotherapy, ultrasound‐guided hydrodistension, interferential therapy, hyaluronic acid injections, and extracorporeal shockwave therapy (ESWT) [22–24]. Electrotherapy modalities reduce pain and enhance functional outcomes through thermal mechanisms, electrical stimulation, and neuromodulation [20, 25]. Concurrently, therapeutic ultrasound utilizes acoustic waves to deliver targeted energy to deep tissues [20, 26]. Hyaluronic acid injections enhance synovial fluid viscosity to minimize friction and restore ROM. Beyond these mechanical benefits, hyaluronic acid exerts a potent anti‐inflammatory effect by suppressing cytokine‐induced responses and reducing synovial inflammation, which synergistically mitigates pain and restores joint function [27, 28].

First introduced for medical use in the 1980s [29], ESWT is a method with proven effectiveness and safety [30, 31], for conditions such as myofascial pain syndrome [32], knee pain [33, 34], chronic pelvic pain syndrome [35], chronic rotator cuff tendinitis [31], sacroiliac joint pain [36], and frozen shoulder [32]. Although its therapeutic mechanism is not fully clarified, ESWT exerts a mechanotransduction effect on soft tissues and bone, inducing neoangiogenesis, growth factor release, and analgesia [37–39]. F‐ESWT is generally safe. Side effects usually depend on the energy level used. Common side effects are minor and temporary. Patients often experience local pain during or after treatment, skin redness, and mild swelling or bruising; less common complications include temporary numbness, tingling, or a short period of increased pain. Severe tissue damage is very rare [40–42].

To date, very few studies have evaluated the use of ESWT for frozen shoulder, and most have addressed only short‐term outcomes (i.e., < 3 months [43]) results. The aim of this study was to evaluate the long‐term (12 month) results of f‐ESWT in the treatment of idiopathic frozen shoulder.

## 2. Materials and Methods

This study was a prospective investigation (Level of Evidence: IV) involving 94 patients who underwent f‐ESWT for idiopathic frozen shoulder between January 2021 and September 2022. For patients with unilateral involvement, frozen shoulder was defined as a passive external rotation of less than 50% compared to the contralateral, unaffected shoulder, in accordance with established criteria [44]. In cases of bilateral involvement, the diagnosis was established by the presence of pain and functional restriction in both active and passive ROM for a duration exceeding 1 month [45]. The study protocol was approved by the local ethical committee and conducted in compliance with the Helsinki Declaration. Written informed consent was obtained from all participants.

The inclusion criteria were as follows: (1) age ≥ 18 years, (2) diagnosis of idiopathic frozen shoulder, (3) intact rotator cuff, and (4) minimum 12‐month follow‐up duration. The exclusion criteria were as follows: (1) rotator cuff tears, (2) rotator cuff calcific tendinopathy, (3) glenohumeral osteoarthritis, (4) history of shoulder trauma or previous surgery, (5) neurological disorders of the upper extremities, (6) neoplastic conditions, (7) a history of alcohol or substance abuse, (8) significant cognitive impairment, and (9) inability to comprehend or complete the required clinical questionnaires. Diagnosis was based on clinical evaluation (medical history, signs, and symptoms) and radiological assessment, including X‐rays and magnetic resonance imaging (MRI). MRI scans were specifically utilized to identify potential rotator cuff tears or any additional intra‐ or extraarticular glenohumeral joint abnormalities that would warrant alternative treatment approaches [46]. Baseline data included patient age, sex, dominant arm, comorbidities, and smoke. Furthermore, we recorded the duration between symptom onset and the initiation of f‐ESWT (categorized as < 3 months or ≥ 3 months) and any prior conservative treatments administered.

### 2.1. Interventions

All patients were fully informed regarding the potential risk and benefits of the proposed treatment, and written informed consent was obtained. The intervention was performed using a PiezoWave2 device (Richard Wolf GmbH, Elvation Medical GmbH, Kieselbronn, Germany). All procedures were carried out by a single medical doctor (ADG), specialized in shock wave therapy, who performed the clinical examination and identified the focal target regions. Each session consisted of 3000 impulses delivered at a frequency of 5 Hz, with an energy flux density (EFD) ranging from 0.10 to 0.15 mJ/mm^2^. The intensity was titrated according to individual patient pain tolerance. During the procedure, patients were placed in the supine position, and standardized coupling gel pads were applied to the target areas. The application protocol followed a specific directional approach. The first 1500 impulses were delivered via an anterior‐to‐posterior direction, with the target zone located approximately 1 cm inferior to the coracoid process. Subsequently, the remaining 1500 impulses were applied in a posterior‐to‐anterior direction, targeting the area beneath the lateral border of the scapular spine. Sessions were conducted at 7‐day intervals, with a maximum of 10 sessions allowed, depending on the patient’s clinical progress and pain relief. To facilitate muscle relaxation, heat packs were applied to the anterior glenohumeral joint for 10 min prior to the commencement of the f‐ESWT.

In addition, all patients adhered to a rehabilitation and home‐based exercise regimen developed by a physiotherapist [47].

### 2.2. Clinical Assessment

Each patient was evaluated using the Constant–Murley Score (CMS), the Disabilities of the Arm, Shoulder and Hand (DASH) questionnaire, and the Visual Analog Scale (VAS). These metrics were recorded at baseline (T0), immediately upon completion of the treatment (T1), and at the 2‐month (T2), 6‐month (T6), and 12‐month (T12) follow‐up intervals. Clinical assessments were conducted by trained physicians who were blinded to the diagnosis and treatment protocols. Furthermore, all patients were monitored for intra‐ and posttreatment complications, as well as for their successful return to work and preinjury sporting activities.

### 2.3. Statistical Analysis

Normality of data distribution was assessed using the Shapiro–Wilk test. Given the distribution, parametric tests were utilized for the analysis. Data were measured and reported with an accuracy of one decimal place. Continuous variables were expressed as mean and standard deviation (SD), whereas discrete variables were presented as median and range; categorical variables were reported as frequencies and percentages. The significance of differences between and among variables was evaluated using Student’s t‐test and ANOVA, followed by Bonferroni post hoc analysis. Univariate linear regression analyses were performed on the entire study population to determine whether the number of f‐ESWT sessions could serve as a predictor for CMS, DASH, and VAS outcomes at T1, T2, T6, and T12. All statistical procedures were performed using IBM SPSS Statistics software (Version 21.0.0.1; IBM Corp., Armonk, NY, USA). A *p* value of less than 0.05 was considered statistically significant.

## 3. Results

Of the initial 94 patients, 17 were lost to follow‐up, leaving 77 patients available for the final evaluation. The characteristics of the study population are summarized in Table 1. There were 42 (54.4%) females and 35 (45.5%) males, with a mean age of 57 ± 9.3 years (ranging 25–87). The median number of performed ESWT sessions was 6 (ranging 2–10). Forty‐three (55.8%) patients underwent ESWT treatment within 3 months from symptom onset, while 34 (44.2%) patients started treatment after 3 months.

**TABLE 1:** Demographic and clinical baseline characteristics of included patients.

Patient characteristics (*N* = 77)	Value
Age at treatment (years), mean (range)	57 (25–87)
Gender, *n* (%)	
Females	42 (54.5%)
Males	35 (45.5%)
Side treated, *n* (%)	
Right	48 (62.3%)
Left	23 (29.9%)
Bilateral	6 (7.8%)
Dominant arm involved	51 (66.2%)
Comorbidities, *n* (%)	
Hypothyroidism	8 (10.4%)
Diabetes mellitus	6 (7.8%)
Pretreatment physical therapy, *n* (%)	
Yes	41 (53.2%)
No	36 (46.8%)
Smoking status, *n* (%)	
No	70 (90.9%)
Yes	7 (9.1%)

Overall, a statistically significant improvement in CMS and a significant decrease in both DASH and VAS values (*p* < 0.001 for all) were observed across measurements, as shown in Table 2. Bonferroni post hoc analysis was also performed. DASH scores significantly decreased from T0 to T1 (*p* = 0.003), T2 (*p* < 0.001), T6 (*p* < 0.001), and T12 (*p* < 0.001); there was no statistically significant difference among T1, T2, and T6, but a statistically significant difference was found between T1 and T12 (*p* < 0.001) (Table 3). CMS significantly improved from T0 to T1, T2, T6, and T12 (*p* < 0.001 for all); no significant difference was observed between T1 and T2, while significant differences were found between T1 and T6 and between T1 and T12 (*p* < 0.001) (Table 4). VAS significantly decreased from T0 to T1, T2, T6, and T12 (*p* < 0.001); no significant difference was found among T2, T6, and T12, though significant differences were observed between T1 and T6 (*p* = 0.012) and between T1 and T12 (*p* = 0.047) (Table 5) (Figures 1–3).

**TABLE 2:** Function scores with ANOVA tests results.

Clinical scores	Pretreatment	Posttreatment	T2	T6	T12	*p* value
DASH Score	43.4 ± 20.0	32.0 ± 17.7	23.7 ± 14.8	22.6 ± 20.8	15.9 ± 15.0	**< 0.001**
CMS Score	43.9 ± 29.0	66.4 ± 18.5	75.1 ± 17.0	82.0 ± 15.2	88.0 ± 14.0	**< 0.001**
VAS Score	7.5 ± 2.0	3.0 ± 2.0	2.3 ± 1.6	1.8 ± 1.2	1.9 ± 1.5	**< 0.001**

*Note:* Mean ± standard deviation (SD); DASH = Disabilities of the Arm, Shoulder and Hand. Pretreatment time (T0), at the end of the treatment (T1), and at 2 months (T2), 6 months (T6), and 12 months (T12) of follow‐up. Significant *p* value (< 0.05). The bold values indicate significant *p* value (< 0.05).

Abbreviations: CMS, Constant–Murley Score; VAS, Visual Analog Scale.

**TABLE 3:** Post hoc analysis for DASH.

Time point (I)	Pretreatment	Posttreatment	T2	T6
Posttreatment	−11.71 (*p* = 00.003) [−20.82, −2.6]	—	—	—

T2	−20 (*p* < 0.001) [−29.04, −10.61]	−8.29 (*p* = 0.131) [−17.69, 1.1]	—	—

T6	−21.16 (*p* < 0.001) [−30.91, −11.41]	−9.54 (*p* = 0.065) [−19.2, −0.29]	−1.15 (*p* = 1.000) [−11.17, 8.86]	—

T12	−27.81 (*p* < 0.001) [−37.88, −17.74]	−16.1 (*p* < 0.001) [−26.16, −6.03]	−7.8 (*p* = 0.333) [−18.13, 0.72]	−6.64 (*p* = 0.785) [−17.29, 4]

*Note:* DASH means Disability of the Arm, Shoulder and Hand. Pretreatment time (T0), the end of the treatment (T1), and at 2 months (T2), 6 months (T6) and 12 months (T12) of follow‐up. Values represent the mean difference (I‐J), followed by the *p* value in parentheses and the 95% confidence interval in brackets. Significant *p* value (< 0.05).

**TABLE 4:** Post hoc analysis for CMS.

Time point (I)	Pretreatment	Posttreatment	T2	T6
Posttreatment	22.42 (*p* < 0.001) [12.82, 32.02]	—	—	—

T2	31.12 (*p* < 0.001) [21.89, 40.34]	8.7 (*p* = 0.121) [−1.04, 18.44]	—	—

T6	38.06 (*p* < 0.001) [28.8, 47.32]	15.64 (*p* = 0.012) [5.86, 25.42]	6.94 (*p* = 0.378) [−2.46, 16.35]	—

T12	44.21 (*p* < 0.001) [34.61, 53.81]	21.79 (*p* ≤ 0.001) [11.68, 31.89]	13.09 (*p* = 0.002) [3.34, 22.83]	6.14 (*p* = 0.766) [−3.63, 15.92]

*Note:* Pretreatment time (T0), the end of the treatment (T1), and at 2 months (T2), 6 months (T6), and 12 months (T12) of follow‐up. Values represent the mean difference (I‐J), followed by the *p* value in parentheses and the 95% confidence interval in brackets. Significant *p* value (< 0.05).

Abbreviation: CMS, Constant–Murley Score.

**TABLE 5:** Post hoc analysis for VAS.

Time point (I)	Pretreatment	Posttreatment	T2	T6
Posttreatment	−4.456 (*p* < 0.001) [−5.33, −3.58]	—	—	—

T2	−5.166 (*p* < 0.001) [‐6.07, −4.26]	−0.710 (*p* = 0.352) [−`1.66, 0.24]	—	—

T6	−5.596 (*p* < 0.001) [−6.54, −4.66]	−1.141 (*p* = 0.012) [−2.13, −0.15]	−0.430 (*p* = 1.000) [−1.44, 0.58]	—

T12	−5.531 (*p* < 0.001) [−6.56, −4.51]	−1.075 (*p* = 0.047) [−2.14, −0.01]	−0.365 (*p* = 1.000) [−1.45, 0.72]	0.066 (*p* = 1.000) [−1.06, 1.19]

*Note:* Pretreatment time (T0), the end of the treatment (T1), and at 2 months (T2), 6 months (T6), and 12 months (T12) of follow‐up. Values represent the mean difference (I‐J), followed by the *p* value in parentheses and the 95% confidence interval in brackets. Significant *p* value (< 0.05).

Abbreviation: VAS means Visual Analog Scale.

**FIGURE 1:**
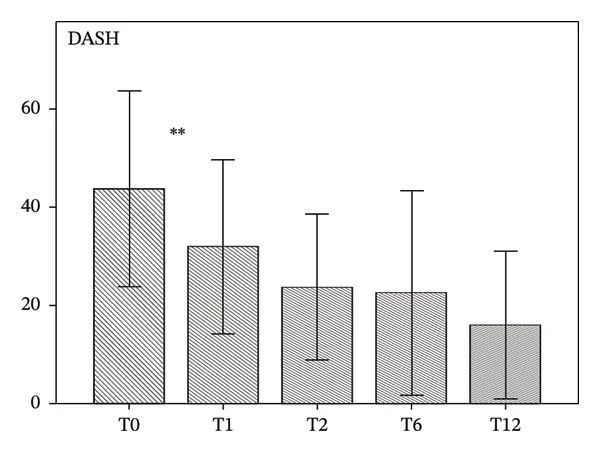
Graphical representation of DASH measurements. DASH means Disability of the Arm, Shoulder, and Hand. ^∗∗^ *p* < 0.01.

**FIGURE 2:**
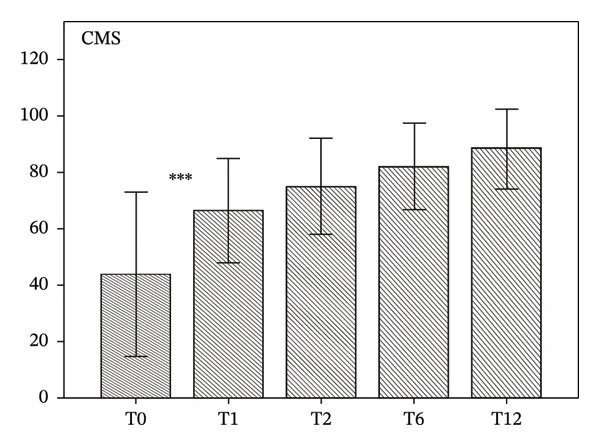
Graphical representation of CMS measurement. CMS means Constant–Murley Score. ^∗∗∗^ *p* < 0.001.

**FIGURE 3:**
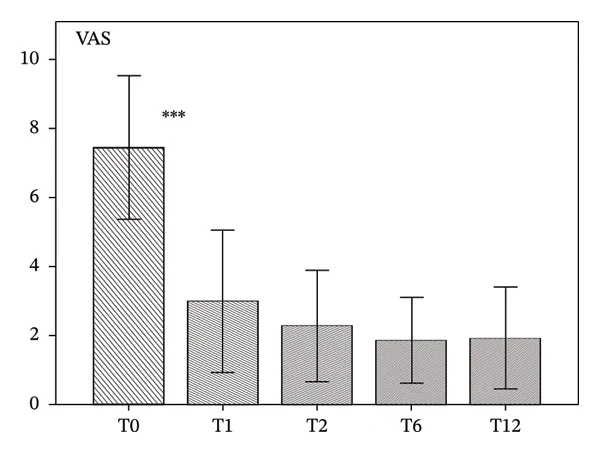
Graphical representation of VAS measurements. VAS means Visual Analog Scale. ^∗∗∗^ *p* < 0.001.

However, no statistically significant differences were detected between contiguous measurements.

Patients who underwent f‐ESWT within 3 months from the onset of symptoms exhibited a statistically significant lower DASH at T2 compared to those who started treatment after 3 months (19.2 ± 11.2 versus 27.8 ± 17.4, *p* = 0.034).

Linear regression analysis revealed that a lower DASH at T2 was associated with a fewer number of performed ESWT sessions (*β* = 0.327, *p* = 0.014). To identify a minimum number of ESWT sessions to be performed to potentially predict better clinical outcome, arbitrary cutoffs were tested, and the best model (as explained by the lowest *p* value detected) is reported, as previously described [48]. Patients who underwent fewer than 7 ESWT sessions exhibited significantly lower DASH at T1 (28.5 ± 16.7 versus 38.5 ± 18, *p* = 0.031) and T2 (20.1 ± 13 versus 30.7 ± 17, *p* = 0.013).

No intra‐ and posttreatment complications occurred, and all patients returned to their work and sports activities.

## 4. Discussion

The aim of this study was to evaluate the 12‐month long‐term [43] results of f‐ESWT combined with physiotherapy in the treatment of idiopathic frozen shoulder. Overall, a statistically significant improvement in CMS and a reduction in DASH and VAS were observed from the baseline to posttreatment evaluations. Detailed serial measurements showed a bimodal trend of clinical improvement; a statistical difference was found from baseline to both the end of the treatment and the 2‐month follow‐up, followed by a clinical plateau leading to a final, further statistically significant improvement at the 12‐month follow‐up. An f‐ESWT protocol consisting of fewer than 7 sessions, started within 3 months of symptom onset, achieved significantly lower DASH in the short term. No complications were observed. All patients returned to their previous level of work and sports activity.

Three distinctive characteristics of the current study are worth addressing. First, f‐ESWT was used. Hameedi et al. [49] compared the effect of radial (r‐ESWT) and focused shockwaves therapy on clinical and functional outcomes. The authors failed to demonstrate differences between the two procedures, although they hypothesized a superiority of r‐ESWT in the short‐term. While r‐ESWT works on a larger superficial area with low energy density, f‐ESWT targets a smaller area with accuracy and depth; all waves were delivered to the tissue depth, with a small spread of the energy. Therefore, it can be hypothesized that f‐ESWT could be more appropriate for achieving a long‐term response, considering its biological effects on tissues [50, 51].

The second major issue in this study was the follow‐up. Almost all studies published to date deal with short‐term (i.e., < 3 months [43]) follow‐up [6, 47, 50, 52, 53], and two studies report the 5‐ and 6‐month follow‐up results of r‐ESWT [11, 21]. To the best of our knowledge, the current study presents the largest case series with the longest follow‐up ever published, regarding the treatment of idiopathic frozen shoulder with f‐ESWT. Both the duration of the final follow‐up and the intermediate evaluations allowed us to clearly detect the trend of clinical results following f‐ESWT: beyond the short‐term improvement, we found a statistically significant improvement in CMS and a reduction in DASH and VAS at the 12‐month follow‐up, thus confirming our hypothesis that f‐ESWT could guarantee a long‐term recovery. In this context, it should be considered that only cases of idiopathic frozen shoulder were included, and it has been previously reported that better functional outcomes after treatment are associated with idiopathic etiology [8]. Similar findings were also reported regarding the reduction of pain [53] in the short‐ and the long‐term period [54]. In contrast, worse scores for pain, satisfaction, and functional activity have been previously reported for posttraumatic and postoperative frozen shoulder cases [55, 56].

As a third major issue, regression analyses were conducted to detect outcome predictors, a topic that has not been explored in previous studies. Notably, we demonstrated that an ESWT protocol consisting of fewer than 7 sessions, started within 3 months of symptom onset, achieved significantly lower DASH scores in the short term. Previous published studied reported protocols ranging from 4 to 12 ESWT sessions [10, 21, 47, 57], but no information regarding outcome variations relative to treatment length had been provided. Our results may be justified by the assumption that longer ESWT treatments could increase proinflammatory cytokines and decrease immunosuppressive T‐regulatory cells, as previously suggested in patients suffering from osteoarthritis [58].

Certain limitations of the present study have to be acknowledged. Given the lack of a control group, potential bias cannot be excluded. Considering the pathophysiology of frozen shoulder and its potential for self‐limitation, a control group undergoing no treatment would have provided an interesting comparison between the rate of spontaneous recovery and functional recovery after f‐ESWT. Moreover, unlike previous articles adopting a standardized protocol, a variable number of f‐ESWT sessions was used in this study, tailored to the clinical features of each patient. We are aware that this choice could introduce bias; however, the “ultracustomization” of treatments is nowadays a prominent topic, and such a tailored approach allowed us to compare different treatment lengths to identify the ideal number of applications for a single patient. Finally, mental status could influence quality of life and functional results, as demonstrated in various procedures [59–62]. The prospective nature of data collection, the use of validated functional assessments, the statistical reliability of the regression analyses, the large study group, and the 12‐month follow‐up represent considerable strengths of the present study.

In conclusion, after a 12‐month follow‐up, a statistically significant improvement in CMS and a reduction in DASH and VAS was observed in patients treated with f‐ESWT for idiopathic frozen shoulder. The procedure was safe and well tolerated by patients. Symptom onset within 3 months and a maximum of 7 sessions may be suggestive of achieving significant improvement of clinical and functional outcomes. Future research should focus on this therapeutic approach to better understand the effect and appropriate protocols.

## Author Contributions

Each author fulfills each of the authorship requirements.

Alfonso Di Giorno, Giorgio Gasparini, and Michele Mercurio conceptualized and designed the study, wrote the paper, and critically revised the manuscript as submitted; Valentina Sanzo and Michele Mercurio performed statistical analysis, interpreted data, wrote the paper, and drafted the final manuscript as submitted; Giovanni Carlisi performed data collection, contributed to the interpretation of data, and wrote the paper; Fabio Catani, Giuseppe Porcellini, and Stefano Gumina contributed to the interpretation of data and wrote the paper; Salvatore Maria Tecce contributed to data collection and wrote the paper; Giorgio Gasparini and Michele Mercurio coordinated data collection and critically reviewed the manuscript.

## Funding

No funding was received for this research.

## Disclosure

All authors read and approved the final manuscript as submitted.

## Ethics Statement

The study protocol was approved by the local Ethics Committee (Magna Graecia University 111/2025), and the research was conducted in compliance with the Declaration of Helsinki.

## Consent

Informed consent for publication was obtained from all individual participants included in the study.

## Conflicts of Interest

The authors declare no conflicts of interest.

## Data Availability

The data that support the findings of this study are available from the corresponding author upon reasonable request.
